# Accuracy and Reliability of Cone-Beam Computed Tomography for Linear and Volumetric Mandibular Condyle Measurements. A Human Cadaver Study

**DOI:** 10.1038/s41598-017-12100-4

**Published:** 2017-09-20

**Authors:** Verónica García-Sanz, Carlos Bellot-Arcís, Virginia Hernández, Pedro Serrano-Sánchez, Juan Guarinos, Vanessa Paredes-Gallardo

**Affiliations:** 1 Department of Orthodontics, Faculty of Medicine and Dentistry, University of Valencia, Valencia, Spain; 2 Department of Anatomy, Faculty of Medicine and Dentistry, University of Valencia, Valencia, Spain

## Abstract

The accuracy of Cone-Beam Computed Tomography (CBCT) on linear and volumetric measurements on condyles has only been assessed on dry skulls. The aim of this study was to evaluate the reliability and accuracy of linear and volumetric measurements of mandibular condyles in the presence of soft tissues using CBCT. Six embalmed cadaver heads were used. CBCT scans were taken, followed by the extraction of the condyles. The water displacement technique was used to calculate the volumes of the condyles and three linear measurements were made using a digital caliper, these measurements serving as the gold standard. Surface models of the condyles were obtained using a 3D scanner, and superimposed onto the CBCT images. Condyles were isolated on the CBCT render volume using the surface models as reference and volumes were measured. Linear measurements were made on CBCT slices. The CBCT method was found to be reliable for both volumetric and linear measurements (CV < 3%; CCI > 0.90). Highly accurate values were obtained for the three linear measurements and volume. CBCT is a reliable and accurate method for taking volumetric and linear measurements on mandibular condyles in the presence of soft tissue, and so a valid tool for clinical diagnosis.

## Introduction

Cone-Beam Computed Tomography (CBCT) has been proved to be an accurate and reliable method for measuring craniofacial structures. Several published studies have assessed its accuracy and reliability by scanning dry skulls in order to compare linear^1–3^ and volumetric measurements^4–7^ taken from physical structures and from CBCT images.

Kayipmaz *et al*. and Sezgin *et al*. demonstrated the accuracy of CBCT for measuring volumes. Both studies applied Cavalieri’s principle to CBCT images and compared the results with physical volume calculations based on the Archimedean principle^6,7^.

Other image modalities may be valid for assessing different structures present in the craniofacial complex such as cartilage. Magnetic resonance (MRI) is often used for this purpose. Previous studies have evaluated MRI for accuracy on cartilage measurements. However, although performed on cadavers, these researches did not compare the digital measurements to the physical ones, making the comparisons with CT images^8,9^.

The morphology and dimensions of the mandibular condyles play an important role in temporomandibular disorders^10^, facial asymmetries^11^ and certain malocclusions^12^, and so their assessment is of the utmost importance in diagnosis. For this reason, an accurate and precise measurement method is crucial.

Some studies of CBCT accuracy have focused on the maxillary bones, while others have specifically studied the mandibular condyles, performing the CBCT scans on dry human skulls^13,14^. However, such studies provide limited information because the soft tissue component is not considered. When soft tissues are present, their attenuation coefficients can decrease the quality of the image, and so the absence of these tissues, replacing them with air, increases contrast and accuracy.

Ganguly *et al*. evaluated the accuracy of CBCT on linear measurements with the soft tissue intact. They used six embalmed heads, which were sectioned to introduce radiopaque markers before taking the CBCT scan. The structures were not extracted to perform the physical measurements, being directly measured on the section with intact soft tissues^15^_._ No other studies have assessed the accuracy of CBCT on linear measurements with the soft tissues present when the CBCT scan was taken. Furthermore, to date, no investigation has analyzed the accuracy of CBCT for volumetric measurement in this context.

For this reason, an analysis of the accuracy of both linear and volumetric CBCT calculations on mandibular condyles within the surrounding soft tissues – in this way resembling clinical conditions – is timely and necessary.

## Objectives

The aims of this study were to evaluate the reliability and accuracy of Cone Beam Computed Tomography (CBCT) for taking linear and volumetric measurements of mandibular condyles with the soft tissues intact.

## Materials and Methods

This study was designed to meet criteria established in the STROBE (Strengthening the Reporting of Observational Studies in Epidemiology) statement and Helsinki declaration guidelines for research involving human subjects. The legal guardian of each cadaver gave informed consent and anonymity has been preserved. The study protocol was approved by the University of Valencia Ethics Committee for Human Research (H1477325246191). All methods were performed in accordance with the relevant guidelines and regulations described in the proposal submitted to the Ethical Committee.

### Sample

Six embalmed cadaver heads with soft tissues intact belonging to 4 males and 2 females (mean age, 72.4 SD 9.2 years) were provided by the Anatomy Department of the University of Valencia and selected applying the following criteria: 1) adult individuals and 2) absence of craniofacial injuries or deformations.

CBCT scans of the six cadaver heads were taken with a Planmeca Promax 3D imaging device (Planmeca, Helsinki, Finland) at 90 kV, with a voxel size of 0.2 mm and a field of view of 18 × 20 cm, being the scan time of 18 seconds. The images were saved in DICOM format in the University’s radiographic database, and a numerical code was given to each scan concurring with the corresponding cadaver head’s identification code.

Both mandibular condyles of each head, 12 in total, were cut at the condylar neck level using a piezoelectric device. The specimens were extracted and soft tissue was carefully removed by the same operator. The mean number of teeth present was 10.17 SD 7.36, range17 and median 13.

### Gold Standard calculations

**Figure 1:**
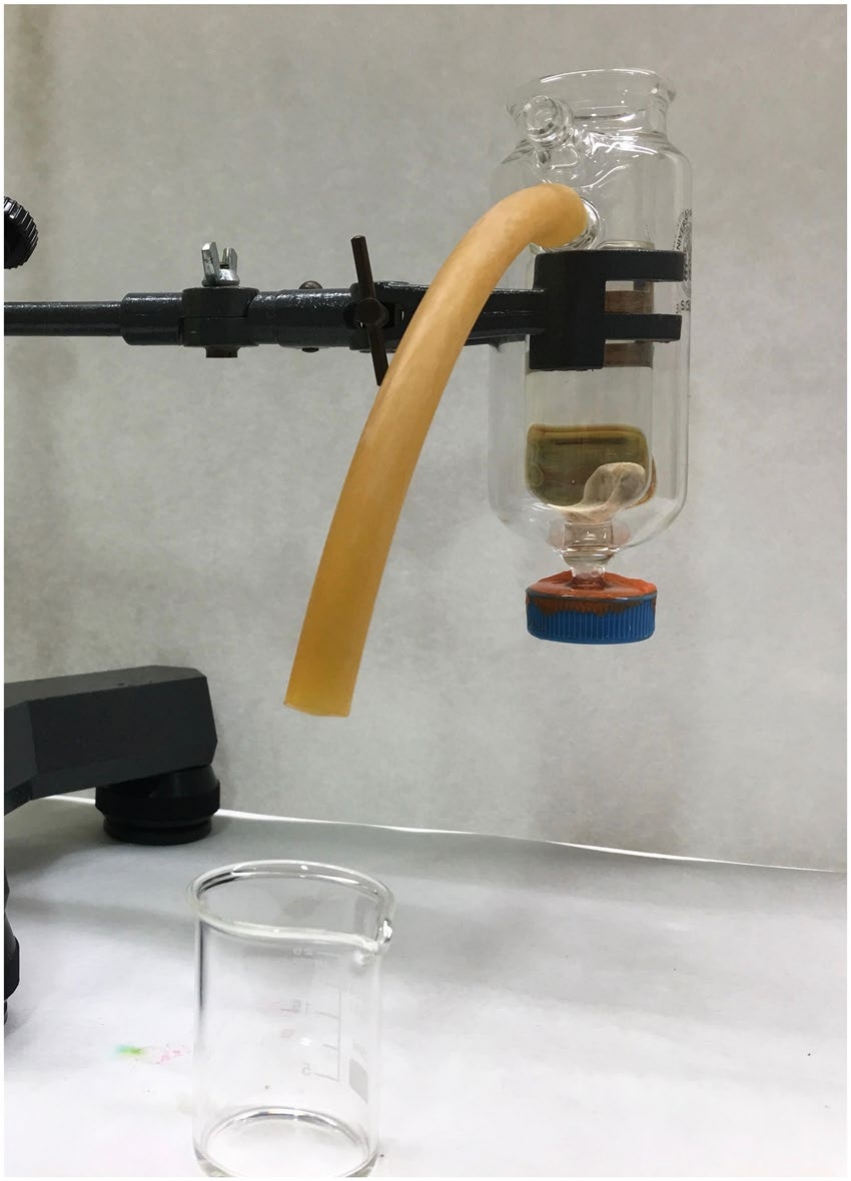
Water displacement method to calculate physical volumes using a pycnometer.

Volumetric and linear measurements were performed on the dry condyles as follows:

For volumetric measurements, each condyle was immersed in a pycnometer, which had been filled with water, and the volume of the run-over water was determined (Fig. 1).

**Figure 2:**
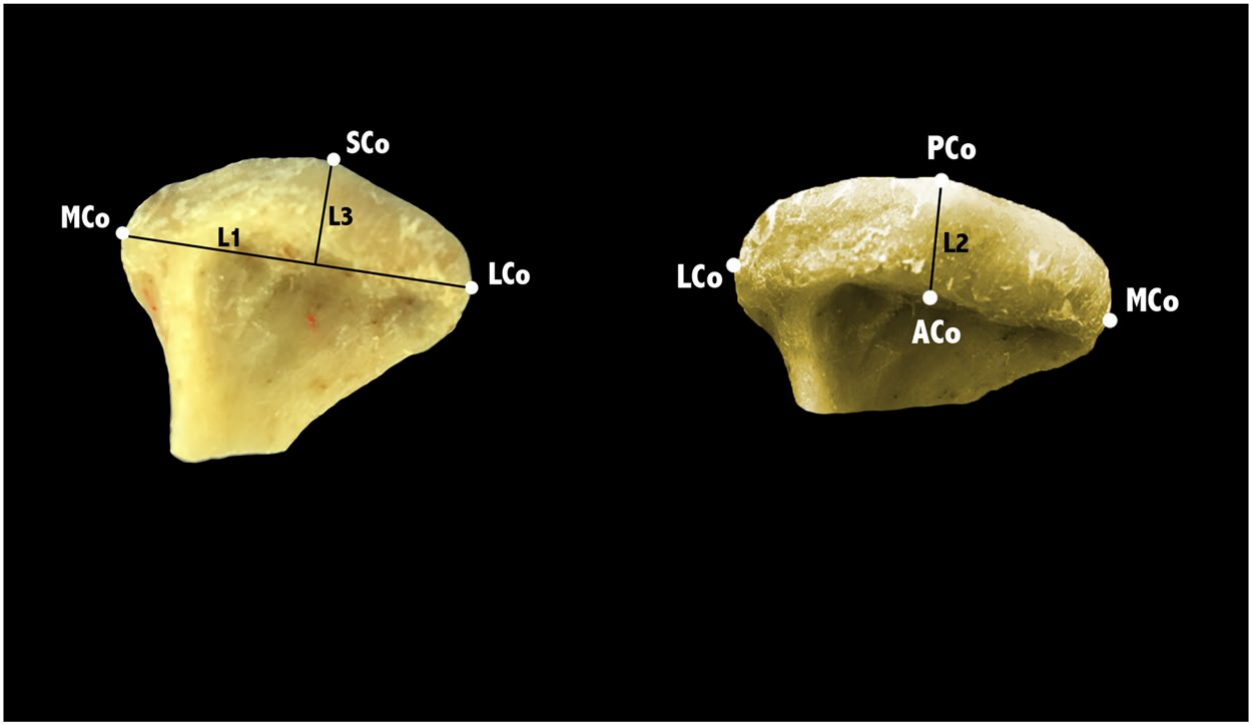
Anatomical landmarks and linear measurements on dry condyles.

For the linear measurements, five anatomical landmarks (Fig. 2) were identified and marked with a pencil on the specimens and three measurements (Fig. 2) were taken using a digital caliper (Precise PS 7215, Burg Wächter, Germany). Anatomical landmarks and linear measurements were defined as follows:

#### Landmarks

- ACo (Anterior): Most anterior extent of the mandibular condyle.
- PCo (Posterior): Most posterior extent of the mandibular condyle.
- LCo (Lateral): Most lateral extent of the mandibular condyle.
- MCo (Medial): Most medial extent of the mandibular condyle.
- SCo (Superior): Most superior aspect of the mandibular condyle.

#### Linear measurements

- L1 (LCo-MCo): Condylar width measured on the coronal view. Linear distance between lateral and medial landmarks.
- L2 (ACo-PCo): Condylar length measured on the axial view. Linear distance between anterior and posterior landmarks.
- L3 (SCo-L1 perpendicular): Condylar height measured on the coronal view. Linear distance of the perpendicular line traced from SCo to L1.

### CBCT calculations

DICOM (Digital Imaging and Communications in Medicine) data were exported from the CBCT scanner program and imported to the Dolphin Imaging^®^ software and 3D reconstructions were made. This software was used for taking both linear and volumetric measurements.

**Figure 3:**
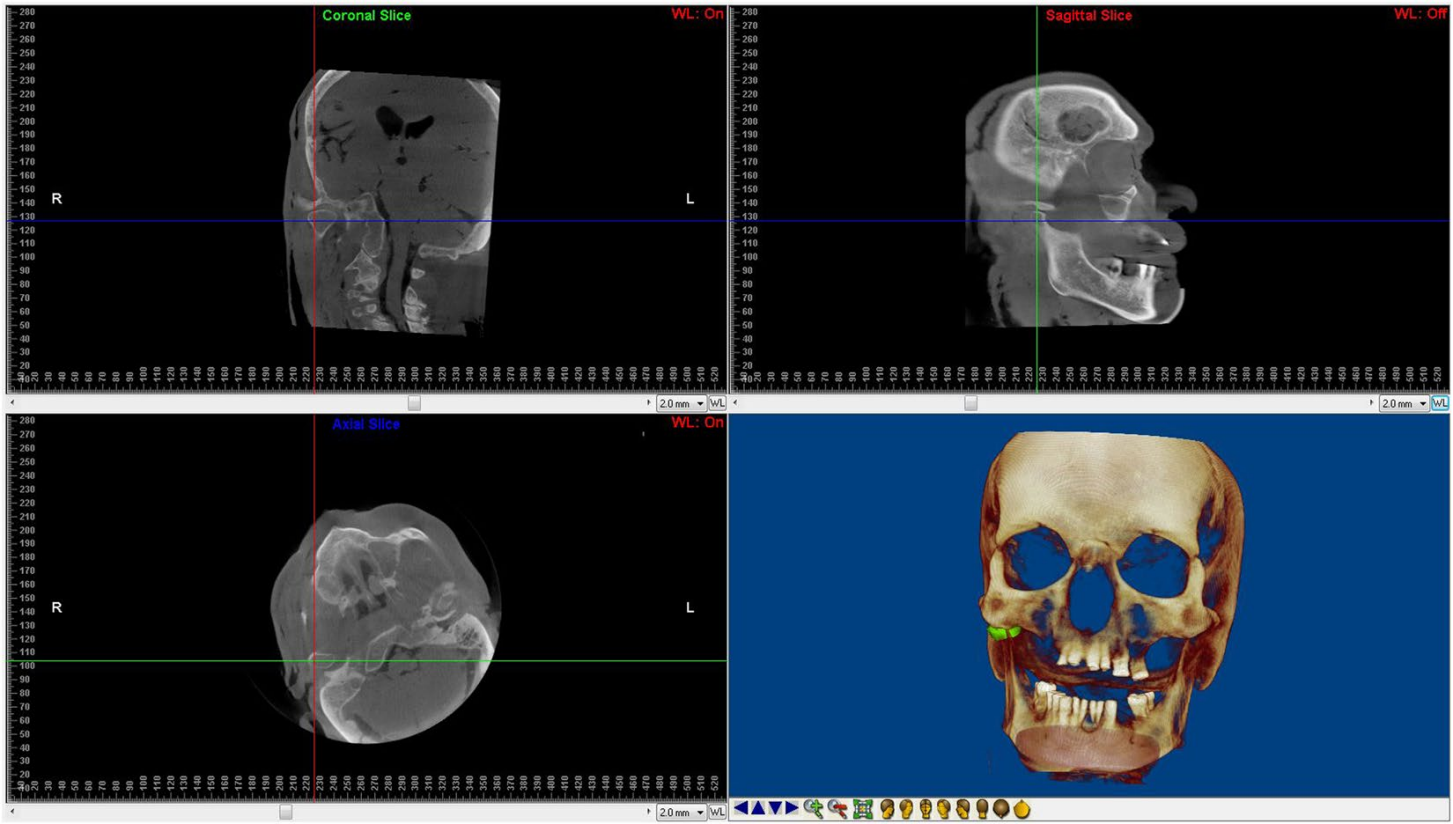
CBCT image orientation and slice selection for linear measurement calculations.

**Figure 4:**
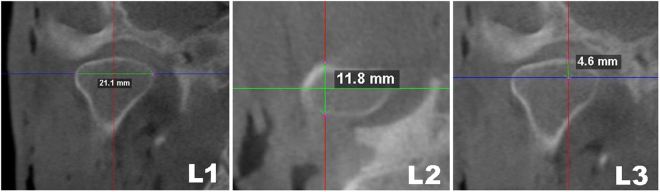
Linear measurements taken on the coronal (L1 and L3) and axial (L2) views of the CBCT image.

For linear measurements, CBCT images were oriented as shown in Fig. 3, with the Frankfurt plane being positioned parallel to the horizontal plane. Three linear measurements (L1, L2 and L3) were then taken (Fig. 4).

**Figure 5:**
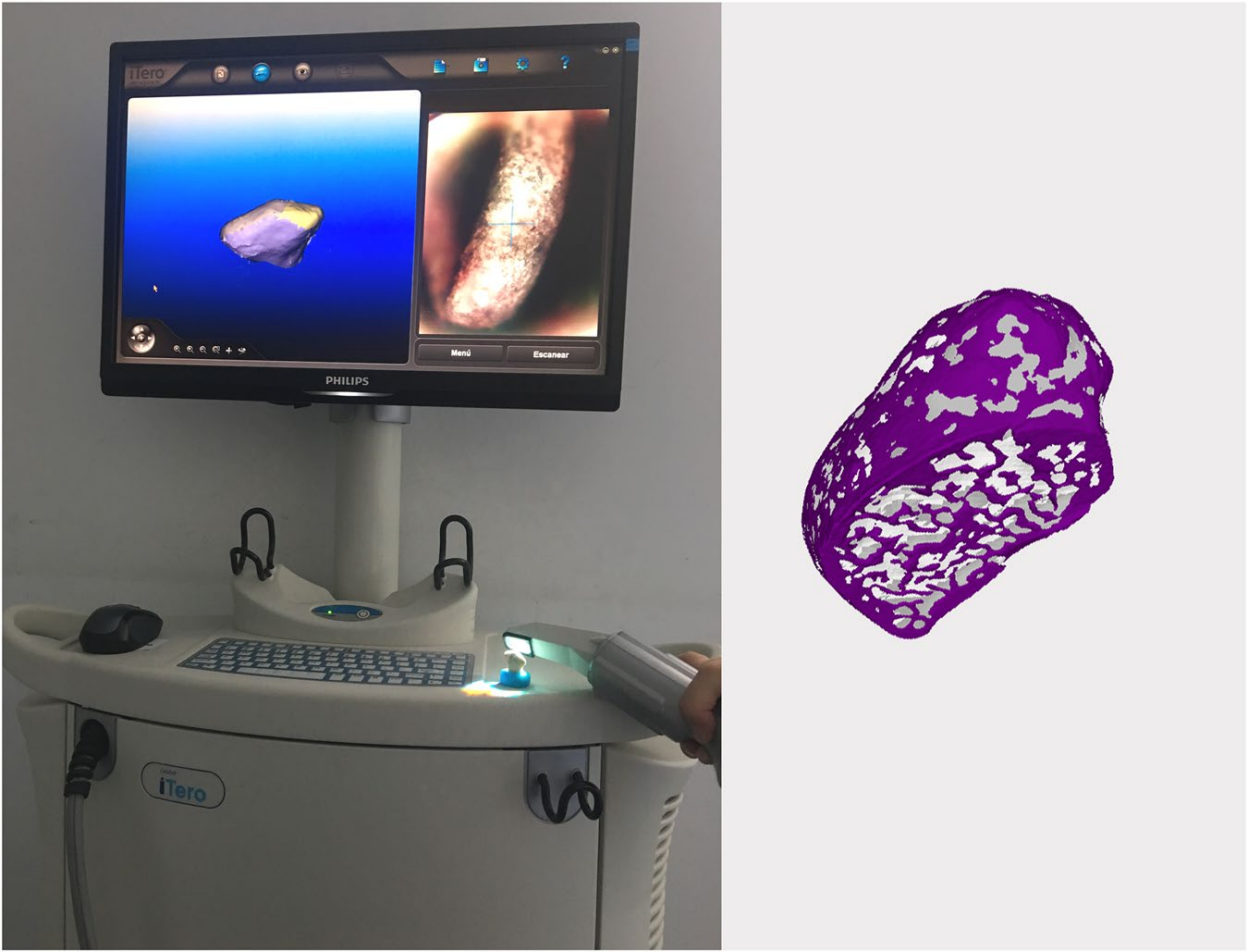
3D scanning procedure using iTero (left) and surface model obtained (right).

**Figure 6:**
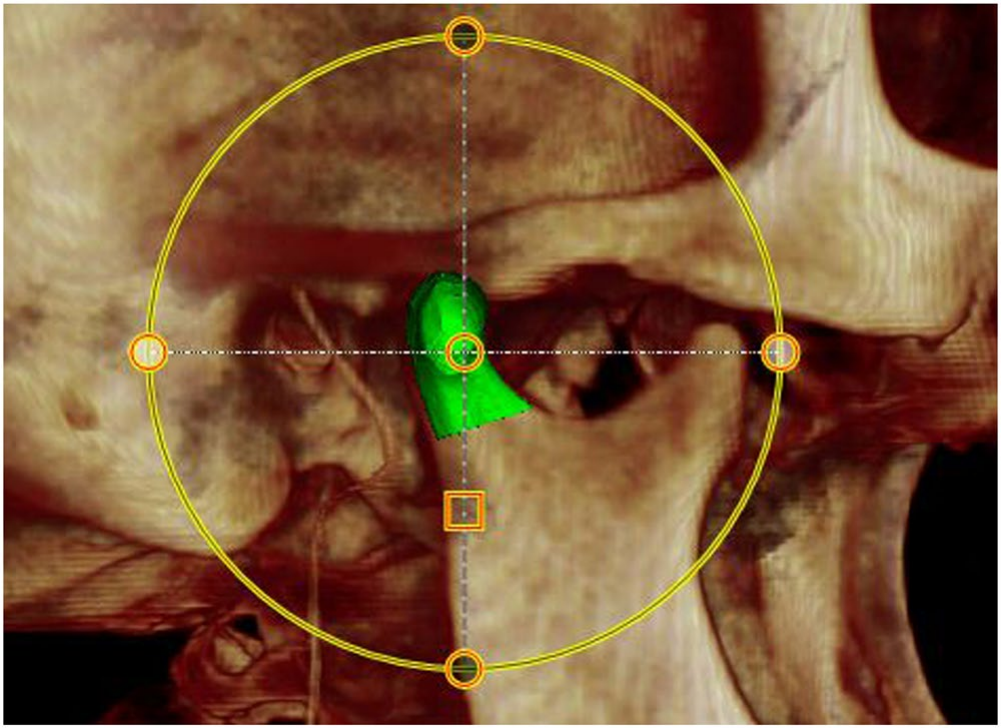
Surface model superimposed onto DICOM reconstruction in Dolphin Imaging® software.

For volumetric calculations, a 3D scanner (iTero; Align Technologies, San Jose, Calif. USA) was used to obtain STL surface models of the twelve condyles (Fig. 5). For the scanning procedure, condyles were mounted on a base made of casting wax. The operator scanned the structure starting at the condyle’s head while the opposing part was inserted into the wax base. Once this part was successfully scanned, the head was inserted in the wax base hence allowing the scanning of the opposing part. Each surface model was imported and superimposed onto its corresponding DICOM reconstruction using the manual superimposition tool included in the Dolphin Imaging® software (Fig. 6). Condyles were isolated on the CBCT render volume using the surface models as reference, to reproduce the exact dry condyle structure. Soft tissues were removed from the CBCT images using the software’s sculpting tool. The volume of the isolated structure was calculated by the software’s automatic volume measuring tool.

Two observers who had been equally trained and calibrated on CBCT measurements were selected to take all CBCT measurements (linear and volumetric). The CBCT calculations were made in six consecutive days. Two condyles belonging to the same head were measured per day.

### Statystical analysis

Method reliability was analyzed by calculating both interobserver and intraobserver error using the Dahlberg formula, Coefficient of Variation (CV%), Paired-t test and Intra-Class Coefficient (ICC). The main observer (VH) took a second set of measurements (all the samples were measured again) one week after the first set of measurements were taken to calculate intra-observer error. The second observer (VG), took all the measurements in the whole sample to calculate inter-observer error.

Accuracy of CBCT measurements was calculated by relating physical and CBCT measurements. T-paired tests and regression models were used. The significance level was set as p < 0.05.

### Data availability

The datasets generated during and/or analyzed during the current study are available from the corresponding author on reasonable request.

## Results

The results are divided into two parts; the first deals with method reliability, and the second with accuracy.

### Reliability

The reliability of the method was found to be high according to both intra and inter-observer error calculations (Table 1). Intra-observer error analysis shows low Dahlberg *d* values, p > 0.05 (t), CV < 2.1% and ICC > 0.97 for all measurements. In the same way, inter-observer error calculations showed homogeneity (p > 0.05), low Dahlberg *d*, CV < 3% and ICC > 0.94 for both volumetric and linear measurements.

**Table 1:** Mean differences and SD between measurements, paired t-test (p-value) and intra/interobserver method error estimators: d Dahlberg, Coefficient of Variation (CV) and Intra-Class Coefficient (ICC) for volumetric and linear (L1, L2 and L3) measurements.

Measurements	Error	Mean	SD	p-value	d Dahlberg	CV (%)	ICC
Volume	Intra	−0.007	0.026	0.359	0.018	1.363	0.99
	Inter	−0.001	0.045	0.950	0.026	1.944	0.99
L1	Intra	−0.001	0.019	0.892	0.013	0.606	0.99
	Inter	−0.008	0.025	0.276	0.011	0.536	0.99
L2	Intra	0.009	0.022	0.174	0.016	1.923	0.98
	Inter	0.009	0.049	0.529	0.022	2.576	0.95
L3	Intra	−0.003	0.017	0.559	0.012	2.070	0.99
	Inter	−0.012	0.044	0.374	0.008	1.408	0.95

### Accuracy

Results of comparisons between CBCT and gold standard measurements assessing the accuracy of the CBCT method are shown in Table 2.

**Table 2:** Mean differences between CBCT and “Gold Standard” volumetric (cm^3^) and linear measurements (mm), Confidence Interval (CI) 95%, paired t-test (p-value), linear regression model results (R^2^ value), slope and intercept.

	Difference CBCT – Gold Standard		CI 95%		p-value	R^2^	Slope [CI 95%]	Intercept [CI 95%]
	Mean	SD	Lower Limit	Upper Limit				
Volume (cm^3^)	−0.010	0.095	−0.071	0.049	0.711	0.922	0.911 [0.724 1.098]	0.110 [−0.150 0.369]
L1 (mm)	−0.004	0.027	−0.022	0.013	0.606	0.981	1.032 [0.931 1.133]	−0.072 [−0.288 0.144]
L2 (mm)	0.019	0.084	−0.035	0.072	0.458	0.702	0.999 [0.541 1.457]	0.020 [−0.368 0.408]
L3 (mm)	0.002	0.054	−0.032	0.037	0.876	0.969	0.768 [0.631 0.906]	0.132 [0.052 0.212]

For volume calculations, the method was found to be highly accurate, with a mean difference between the methods of 0.010** ± **0.095 cm^3^ and strong linear correlation (R^2^ = 0.922). Regarding linear measurements, accuracy was also high, with mean differences between CBCT and gold standard of 0.004** ± **0.027, 0.019 ± 0.084 and 0.002 ± 0.054 cm for L1, L2 and L3 respectively. Linear correlations were strong for L1 and L3 (R^2^ = 0.981 and 0.969 respectively), but weaker for L2 (R^2^ = 0.702). L3 showed a tendency for CBCT to overestimate the measurements when the distances measured were small. Homogeneity was acceptable for all measurements (p > 0.05), which, together with linear correlation results, affirms the accuracy of the CBCT method.

## Discussion

The present study used a small sample size compared to *in vivo* CBCT studies, but acceptable in terms of studies performed using cadavers, with a sample size in accordance with other similar studies^1,3,6,13,15^.

Intra and inter-observer error were calculated, showing that the method was highly reliable. Some studies of bone volume or linear measurements using CBCT have omitted to calculate method reliability^3,6,16^, while others have included analyses of this parameter. Fernandes *et al*. conducted a study of dry mandibles to assess linear measurements with results that concur with the present study, concluding that the measurements taken from CBCT volume rendering were reliable^1^. Unlike their work, the present study performed the linear measurements on CBCT sections rather than 3D reconstructions. Leung *et al*. also found high reliability when measuring alveolar bone defects^2^. Bayram *et al*. assessed the reliability of condyle volume measurement finding high reliability. Unlike the present study, the authors used the Cavalieri principle^13^^.^

CBCT is a good tool for assessing craniofacial structures. Several *in vivo* studies have focused on the anatomy of the condyle to establish relationships with different parameters. Tecco *et al*. studied the volume and surface of mandibular condyles, finding high anatomical variability^17^. Saccuci *et al*. also assessed the volume of the condyles and identified associations between volume and skeletal class^12^. However, in these studies, the accuracy of the measurements was uncertain and cannot be confirmed. To assess method accuracy, it is necessary to use the real structure as the gold standard. But few studies have used cadaver samples to make measurements of mandibular condyles in order to investigate measurement accuracy. In the present study, CBCT images of the structures were measured and compared to the anatomical truth in order to assess the accuracy. It was concluded that the method was highly accurate for volume measurements (R^2^ = 0.922). Bayram *et al*. also investigated the accuracy of volume measurements on nine mandibular condyles, obtaining similar results^13^.

Regarding linear measurement, the present study found the three measurements to be accurate, although the association was weaker for condylar length (L2). In agreement with the present findings, Hilgers *et al*. found CBCT to be remarkably accurate for all three linear dimensions^18^. A study conducted by Schlueter *et al*. assessed accuracy at different levels of density, concluding that the method was accurate, especially when accomplishing 3D reconstruction at low densities^14^.

The studies cited above differ from our research due to the absence of the soft tissues when specimens were scanned. Soft tissues have attenuation coefficients that can affect the x-ray beam passing through the structures, which can compromise image quality^2^. In the present study, CBCT scans of cadaver heads were captured with the soft tissues intact in order to overcome this limitation, and to reproduce real clinical situations as closely as possible. This required a novel protocol as to date no other study has attempted to evaluate both volume and linear dimension accuracy in the presence of the soft tissue component. In order to isolate the condyle in CBCT images so that they would coincide with the actual structures extracted later from the cadaver, surface volumes and superimpositions were performed. A work by Ganguly *et al*. is the only study that has analyzed the accuracy of CBCT on bone measurements in the presence of soft tissues although the study did not focus on condyle structures and did not include volume measurements. The methodology of their research highly differs from the present study since they alter the structures by sectioning them to introduce markers before taking the CBCT^15^. Nevertheless, the study obtaining high measurement accuracy, like the present study.

Overall, studies evaluating accuracy of CBCT on linear and volumetric bone measurements have found this diagnostic tool to be accurate in this regard. However, CBCT has not been checked for accuracy when analyzing other important structures in the temporomandibular complex such as the articular cartilage. Further studies following the protocol of the present research may be of interest to evaluate the accuracy of CBCT on evaluating the cartilage morphology.

The present study analyzed condyles as they are one of the most important parts of the temporomandibular complex, so that condyle morphology often provides a better understanding of certain disorders^19^, and is crucial to accurate diagnosis. One limitation of the present study was that CBCT accuracy was not assessed when applied to the measurement of condylar defects. A work by Honda *et al*. analyzed bony defects in 21 temporomandibular joint autopsy specimens both macroscopically and in CBCT images, finding CBCT to be accurate and reliable^20^. Other studies have assessed accuracy by simulating osseous defects using real bone^21,22^ or acrylic blocks^22^. Patel *et al*. concluded that defects smaller than 2mm are difficult to detect from 3D images^21^. Pinsky *et al*. created artificial defects in both acrylic and real human mandibles, obtaining good accuracy values when diagnosing with CBCT, although the results were more accurate when measurements were taken from the acrylic samples^22^.

Another limitation of the present work is that markers could not be used to identify the landmarks in the CBCT images. By adding markers, the measurements would have been more accurate. However, the fact of maintaining the soft tissues intact, which was one of the strong points of our study, did not allow the introduction of any markers.

Further studies using real bone specimens with the soft tissues intact are needed to thoroughly assess mandibular condyle erosions and defects. These studies will require large sample sizes including a wide variety of abnormalities.

The present study adopted a novel protocol to compare 3D virtual images with real anatomical structures including the soft tissue component for the first time. This study design may benefit future research aiming to reproduce clinical conditions.

## Conclusions

The reliability of CBCT imaging for taking linear and volumetric measurements was found to be high.

CBCT is an accurate method for making both volumetric and linear measurements of mandibular condyles in the presence of the soft tissue component.
